# The Danish National Database for Asthma: establishing clinical quality indicators

**DOI:** 10.3402/ecrj.v3.33903

**Published:** 2016-11-08

**Authors:** Susanne Hansen, Benjamin Hoffmann-Petersen, Asger Sverrild, Elvira V. Bräuner, Jesper Lykkegaard, Uffe Bodtger, Lone Agertoft, Lene Korshøj, Vibeke Backer

**Affiliations:** 1 Research Centre for Prevention and Health, Rigshospitalet Glostrup Hospital, Glostrup, Denmark; 2 Hans Christian Andersen Children’s Hospital, Odense University Hospital, Odense, Denmark; 3 Respiratory Research Unit, Department of Respiratory Medicine, Bispebjerg Frederiksberg University Hospital, Copenhagen, Denmark; 4 Department of Occupational and Environmental Medicine, Bispebjerg – Frederiksberg Hospital, Copenhagen, Denmark; 5 Research Unit of General Practice, Institute of Public Health, University of Southern Denmark, Odense, Denmark; 6 Department of Respiratory Medicine, Naestved Hospital, Region Zealand, Denmark; 7 Department of Respiratory Medicine, Zealand University Hospital Roskilde, Region Zealand, Denmark; 8 Institute of Regional Health Research, University of Southern Denmark, Odense, Denmark; 9 The Danish Clinical Registries, Aarhus, Denmark

**Keywords:** asthma, clinical indicators, quality of care, database, prescription data, hospital data

## Abstract

Asthma is one of the most common chronic diseases worldwide affecting more than 300 million people. Symptoms are often non-specific and include coughing, wheezing, chest tightness, and shortness of breath. Asthma may be highly variable within the same individual over time. Although asthma results in death only in extreme cases, the disease is associated with significant morbidity, reduced quality of life, increased absenteeism, and large costs for society. Asthma can be diagnosed based on report of characteristic symptoms and/or the use of several different diagnostic tests. However, there is currently no gold standard for making a diagnosis, and some degree of misclassification and inter-observer variation can be expected. This may lead to local and regional differences in the treatment, monitoring, and follow-up of the patients. The Danish National Database for Asthma (DNDA) is slated to be established with the overall aim of collecting data on all patients treated for asthma in Denmark and systematically monitoring the treatment quality and disease management in both primary and secondary care facilities across the country. The DNDA links information from population-based disease registers in Denmark, including the National Patient Register, the National Prescription Registry, and the National Health Insurance Services register, and potentially includes all asthma patients in Denmark. The following quality indicators have been selected to monitor trends: first, conduction of annual asthma control visits, appropriate pharmacological treatment, measurement of lung function, and asthma challenge testing; second, tools used for diagnosis in new cases; and third, annual assessment of smoking status, height, and weight measurements, and the proportion of patients with acute hospital treatment. The DNDA will be launched in 2016 and will initially include patients treated in secondary care facilities in Denmark. In the nearby future, the database aims to include asthma diagnosis codes and clinical data registered by general practitioners and specialised practitioners as well.

Asthma is one of the most prevalent chronic respiratory diseases in children and young adults, affecting an estimated 300 million people worldwide (1). The disease is associated with increased morbidity and reduced quality of life, and the costs associated with this disease in the form of health care and sick-leave compensation are significant (2, 3).

Although the disease has been known for centuries, a gold standard diagnostic test for asthma is lacking. In a specialist setting, the cases are typically characterised by respiratory symptoms and a positive asthma challenge, whereas primary care often diagnoses asthma solely based on symptoms, and possibly also on the effect of asthma medication. Previous studies have suggested that undiagnosed cases, dubious asthma diagnosis, and poor asthma control are common (4, 5).

The Danish National Database for Asthma (DNDA) will be launched in 2016 with the overall aim of collecting data on all patients treated for asthma in Denmark and monitoring asthma occurrence, the quality of diagnosis, and management, and has been described in detail elsewhere (6). The database is based on existing health care registers within the Danish health care system, including the National Patient Register, the National Prescription Registry, and the National Health Insurance Services register. Because the Danish health care system is universal and free of charge for everyone, all patients with asthma that are diagnosed or treated are therefore potentially included in the database. The data collected in the DNDA encompass monitoring the trends in the following quality indicators: first, conduction of annual asthma control visits, pharmacological treatment, forced expiratory volume in 1 sec, forced vital capacity (FVC), and asthma challenge testing; second, tools used for diagnosis in new cases and the proportion of skin prick tests performed or the measurement of specific immunoglobulin E concentrations in new patients; and third, annual assessment of smoking status, height and weight measurement, and the proportion of patients with acute hospital treatment (6).

This paper is not a systematic review but presents evidence from the existing literature that has formed the basis for the discussions on the selection of nine quality indicators taken by the steering committee of the DNDA embracing specialists in respiratory medicine, paediatrics, epidemiology, and general practice, representing all regions in Denmark. Due to uncertainty associated with an asthma diagnosis in young children and due to an overlap between symptoms of asthma and chronic obstructive pulmonary disease (COPD) in older patients, the steering committee has decided that the indicators in the DNDA will evaluate the quality of care in asthma patients aged between 6 and 45 years and, therefore, this paper presents literature focusing on this age range.

## Indicator 1: control visits

### Introduction

Asthma control is the degree to which the manifestations of asthma are minimised by therapeutic interventions. The goal of asthma control is two-fold: First, the goal is to reduce the frequency and intensity of symptoms that the patient is currently experiencing or has recently experienced; Second, the goal is to reduce the risk of asthma exacerbations or of progressive loss of lung function over time. Level of asthma control is achieved in response to treatment and determines if the patient’s medication should be adjusted.

### Effect of inhaled corticosteroids in asthma control

In a large randomised study, the Gaining Optimal Asthma ControL study with asthma control as the primary endpoint, almost 60% of patients achieved disease control within the first 8 weeks of the start of, or increase in, inhaled corticosteroids (ICS) (7). Asthma control was defined by no or few day-time symptoms, no or little use of beta-2-agonists, morning peak expiratory flow (PEF) ≥80% of usual or normal PEF, no night-time wakening due to asthma symptoms, no exacerbations, no emergency room visits, or no treatment-related adverse events leading to changes in treatment. Patients were followed over 52 weeks with gradual up-titration of ICS every 12 weeks (to a maximum of fluticasone 500 µg 1×2), followed by a phase of fixed-dose ICS. The overall proportion of patients with well-controlled asthma increased significantly within the 52-week period. Furthermore, in another randomized controlled study (RCT) using inhaled ICS in fixed combination with long-acting beta2-agonist (ICS/LABA), both as maintenance and reliever therapy single maintenance and reliever therapy (SMART), the gain of asthma control has been examined. SMART achieved greater improvements in inflammatory biomarkers, symptom scores, and reliever use in patients treated with ICS alone with uncontrolled symptoms at the time of enrolment. Furthermore, SMART has also been found to prevent development of severe exacerbations (8) and increased adherence (9).

### Frequency of asthma control visits

Global Initiative for Asthma (GINA) guidelines suggest that the evaluation of asthma control is approached by assessing two areas (10): first, assessment of the degree of symptom control, and second, the assessment of risk factors for poor asthma outcome. However, there are no clear recommendations regarding the frequency of patient follow-up. The initial frequency should be determined by the time-course of improvement in different asthma control outcome variables. Asthma symptoms have been shown to be reduced relatively quickly after commencing ICS treatment. Night-time symptoms are the first to disappear within a few weeks, and day-time symptoms typically subside after the first few months (11–13). The greatest effect on forced expiratory volume in 1 sec (FEV1) can be expected within 4 weeks (14, 15), but there will be further improvement in FEV1 around 12–14 weeks after starting treatment (16).

Patients with an asthma diagnosis should be monitored with regular check-ups to ensure adequate symptom control, ensure normal activity level, minimise the risk of exacerbations, ensure normal lung function and lung development in children, and minimise side effects of the medication (10). When exclusively assessing symptom control, the GINA guidelines suggest that adult patients should be controlled around 8–12 weeks after the onset of increased dose of ICS, in order to assess symptoms, the use of short-acting beta agonists (SABA), and lung function (10).

### Adherence and inhalation technique

Poor adherence to therapy is a key problem in asthma management, as poor adherence may have implications for the development of lung function, poor symptom control, and increased risk of hospitalisation (17–19). Up to 24% of exacerbations, and 60% of exacerbations requiring hospitalisation, can be attributed to poor adherence to controller medication (20).

A recent retrospective register study from 2015, including 69,000 participants, reported that almost 20% of first-time prescribed asthma patients did not redeem their ICS prescription (21). The same study showed that within a period of 12 months, the daily requirement of ICS was only covered approximately 19% of the time. This result is in agreement with a recent review of the literature from 2015, where adherence was reported to be between 22 and 63% in the included studies (20).

Adherence has been shown to improve after various types of patient interventions, but no significant effect on clinical endpoints has been reported. Thus, there is currently no evidence suggesting that regular follow-up alone would have a possible positive effect on adherence. In children, there is evidence that adherence to ICS is a strong predictor for long-term asthma control (22).

The potential errors using various inhalers are many (23); over half of the patients, especially the elderly (24) and young children (25), have poor inhalation techniques that, at worst, can result in poor asthma control (26, 27). Most people can learn to use an inhaler correctly after instruction, but after only a few weeks, the number of patients who make mistakes in the use of inhaler will typically increase if techniques are not demonstrated again (28, 29). Written and oral instructions for using the inhaler improve inhalation techniques, but this would be further improved by a physical demonstration by trained personnel (29). Instruction on the use of the inhaler and regular training in its use is shown to reduce errors in the use of inhalers, increase asthma-related quality of life, and reduce PEF variation (30).

## Indicator 2: the use of short-acting beta agonist and asthma control

### Introduction

The pharmacological treatment of asthma is divided into relievers [inhaled SABA, inhaled ICS, inhaled LABA, and oral leukotriene receptor antagonists (LTX)]. The SABA and LABA stimulate the beta-2 receptors, thereby preventing bronchoconstriction, whereas the preventive effect of ICS and LTX works through anti-inflammatory mechanisms. The long-term goal of asthma treatment is to ensure good symptom control and normal activity, minimising the risk of acute exacerbations and the possibility of developing irreversible lung function, while ensuring the least side effects from the treatment (10).

The treatment of both children and adults is based on a phased approach to the patient’s symptoms, risk profile, and needs of SABA. Monotherapy with SABA in adults, adolescents, and children aged 6–11 years is used only for individuals with symptoms less than two times a week, never against nocturnal symptoms, and is not used as the sole remedy for exacerbations. According to GINA, patients who are well controlled and who are not at risk for future exacerbations should be treated with SABA alone while all patients with frequent symptoms should start treatment with ICS.

Despite international guidelines for treatment of asthma, not all patients are treated successfully and in accordance with international guidelines. In a recent cross-sectional study of adult asthma patients, the authors reported that 67% of a group of 493 patients with asthma were not objectively diagnosed with the disease or were undertreated, and only 24% who should be treated with ICS according to GINA guidelines were actually treated with ICS (5). These results are in agreement with similar studies of patients with persistent asthma from Europe, the United States, and Asia that have reported the proportion of patients who are underdiagnosed or undertreated to vary between 48 and 86%, and the proportion of patients who do not receive treatment to be around 35–84% (17, 31–35). The same pattern of inappropriate treatment of asthma has been observed in children where lack of treatment with ICS and inappropriate treatment with oral SABA not recommended by GINA are common (36, 37). However, in children, a diagnosis is often confirmed by treatment with medications, which may explain part of the apparent over-treatment.

The consequence of insufficient treatment not meeting clinical recommendations includes poor asthma control (35, 38) and an increased risk of frequent and severe asthma exacerbations (39, 40). In Denmark, Backer et al. demonstrated that the proportion of patients with uncontrolled asthma was 62%, and this typically included patients with severe asthma requiring asthma treatment step 4 or step 5 according to GINA (35, 41). Several epidemiological studies show that the proper use of ICS paralleled by low SABA consumption is negatively associated with asthma-related hospitalisations and mortality; thus, SABA overuse is associated with an increased risk of serious exacerbations and higher asthma-related health care costs (42–44). There is a need for improved monitoring of the use of asthma medications and of the effect of initiated treatment.

### Consumption of SABA as a measure of the degree of asthma control

The degree of asthma control can be assessed using validated questionnaires (45–47), or by monitoring consumption of SABA as proxy for asthma control, since high consumption of SABA is an expression of poor symptom control, as indicated in previous studies (43, 48–50).

The GINA guidelines define SABA overuse as consumption of more than one SABA container with 200 doses per month based on the study by Suissa et al. having mortality as the major endpoint. The study included 12,301 patients aged 5–54 years who, over a 10-year period, had retrieved at least 10 prescriptions for asthma medications. Mortality increased significantly with consumption of more than 1.7 (95% CI 1.4–2.5) containers with 200 doses per month, which corresponds to 12 SABA containers per year, or about 10 puffs per day (51).

Other studies have similarly studied associations between the use of SABA and asthma control. Davidsen et al. reported a weak correlation between high consumption of SABA, >450 defined daily dose (DDD)/year, and poor symptom control in individuals with and without prophylactic treatment with ICS. This correlation was not significant for consumption of SABA <400 DDD/year (38). Andrews et al. explored the ICS/(ICS+SABA) ratio in 19,512 asthmatic children aged 2–18 years retrospectively and found that a ratio of <0.5 significantly predicted emergency room visits and/or hospitalisations (52). Silver et al. also conducted a retrospective cohort study including 93,604 patients aged 6–56 with asthma who were prescribed asthma medication. It was found that consumption of ≥600 doses of SABA over a 3-month period was associated with an increased risk of emergency room visits and hospitalisation with exacerbation of asthma (53). Paris et al. conducted a similar study in 2008 including 2,056 patients and found a significant positive association between the use of SABA nebulisers and asthma-related emergency room visits and admissions, but no associations with non-nebulisers (48). Finally, an American study found that consumption of more than 3–6 single containers of SABA per year was associated with an increased risk of exacerbations and poorer asthma control and that this risk increased progressively with consumption of >7 single containers per year (54). They later compared various surrogate markers as predictors for asthma exacerbations, and concluded that the number of individual containers with SABA provided to the patient was most useful for predicting asthma outcome followed by the ICS/(ICS+SABA) ratio (55).

## Indicator 3: long-acting beta-agonist use without concomitant ICS

### Introduction

The first inhaled beta2-agonists became available in the late 1940s and contained isoproterenol, which is an agonist for both beta1 and beta2 receptors. In the 1960s, there was an epidemic of asthma-related deaths, which was linked to the use of inhaled isoproterenol. A new epidemic in the late 1970s was related to the use of fenoterol, an inhaled short-acting beta2-agonist. The LABAs salmeterol and formoterol were developed in the late 1980s. With history in mind, there has been a continuous focus on the safety of LABA in asthma patients.

### Evidence of increased mortality associated with LABA as monotherapy in patients with asthma

In 2014, a Cochrane review comparing the incidence of serious adverse events (SAEs) and asthma-related deaths in patients receiving treatment with salmeterol and formoterol compared with placebo treatments was released (56). Data were analysed both as a categorical effect (salmeterol and formoterol together) and as individual effects of salmeterol and formoterol alone. It was concluded that patients treated with LABA (salmeterol and formoterol combined) had an increased risk of SAE and asthma-related deaths compared with placebo. The results were primarily driven by the salmeterol Multicenter Asthma Research Trial from 2006 (57) which was interrupted due to higher mortality and increased incidence of life-threatening asthma-related episodes in the group of patients treated with salmeterol. Analysis of the data from this multi-centre study showed that the increased frequency of deaths and life-threatening asthma-related episodes was significantly higher in the group of African Americans without concomitant ICS therapy (57).

There is still some debate about the safety of the use of LABA+ICS versus ICS alone. Data have not confirmed an increased risk in mortality or SAE for LABA in addition to ICS. Therefore, it cannot be definitely ruled out that treatment with LABA in addition to ICS represents a risk, and additional data are required (56). Large studies assessing the safety of LABA+ICS versus ICS alone prompted by the US Food and Drug Administration are presently being implemented.

GINA further recommends that, if LABA is indicated, LABA should be replaced with a combination product including LABA and ICS in one inhaler to minimise the risk of a situation where the patient is only compliant with the LABA. However, no data have been identified that support this statement.

### Evidence regarding LABA as monotherapy in children

Although several of the abovementioned studies included data on LABA treatment in children, there are generally too few data available to enable meaningful conclusions on the potential increased risk of SAE and mortality during monotherapy with LABA in children (58, 59). Recommendations for children are therefore extrapolated from the adult population.

### The use of anticholinergics in patients with asthma

There is no evidence of increased numbers of SAE or mortality in adult patients using short-term acting anticholinergics (60–62) or in adolescents ≥12 years of age (63).

Historically, anticholinergics have not been used to the same extents as beta2-agonists in asthma. Short-acting anticholinergics (SAMA) may well have shown a significant but modest bronchodilator and symptom-reduction effect in asthma (60); however, this is estimated to be inferior to beta2-agonists (64). In a meta-analysis of studies comparing SAMA/SABA to SABA alone, there were no improvements in symptoms, lung function, or exacerbation rate with the addition of anticholinergics (60).

A short-term study examined the safety and efficacy of long-acting anticholinergics (LAMA) using umeclidinium as monotherapy in asthma and found a modest benefit of umeclidinium over placebo in terms of FEV1 area equal to 60–90 mL (62). The study did not report prior medication use and was too short to address exacerbation risk.

Finally, a study on patients with inadequate disease control on regular ICS use reported that the addition of tiotropium to ICS had greater effect on lung function and asthma symptoms compared with doubling of ICS dose (65). Later studies have confirmed that treatment with anticholinergics as an add-on to ICS or ICS+LABA improves lung function and reduces the number of exacerbations, emergency room visits, and hospitalisations (66, 67).

## Indicator 4: lung function

### Introduction

Lung function measurements, including registration of FEV1 and FVC, constitute the cornerstone of out-patient visits for asthma patients. There are no absolute contraindications, and only a few relative contraindications, including a recent acute coronary syndrome and circumstances which might affect the validity of the measurements, for example, pain, dementia, and incontinence.

### Validity of spirometry

Spirometry is a standardised and validated measurement. FEV1 is typically reported as a percentage of the predicted value (% predicted), based on normal materials collected and stratified for age, sex, height, and ethnicity. It is recommended that the staff carrying out spirometry has undergone relevant training, and they are offered ongoing maintenance of skills (68). Variability of repeated measurements of FEV1 is expected to be maximally 150 mL. The variation over time is much larger, and FEV1 in healthy subjects can vary up to 12% from week to week, and up to 15% from year to year (69, 70).

### The relationship between FEV1, symptoms, and other disease markers

The relationship between FEV1 and severity of symptoms assessed by standardised questionnaires is low (71, 72), which is also the case for PEF (73). In addition, quality of life, as well as inflammation markers, only correlates weakly with FEV1 (74). In contrast, a low FEV1 % predicted is an independent marker for increased mortality, increased risk of exacerbations, as well as increased risk for the development of irreversible airway obstruction (75).

Low FEV1 % predicted has been found to be an independent marker (after adjustment for sex and smoking) for increased risk of exacerbations requiring emergency room visit or hospitalisation (76–79). Osborne et al. has shown that patients with FEV1 of 60–80% predicted had a two-and-a-half times increase in the relative risk of hospitalisation, compared with asthmatic patients with FEV1 ≥80% predicted. Patients with FEV1 <60% predicted had an even higher relative risk (76).

### FEV1 over time in patients with asthma

Overall, patients with asthma have lower lung function than those without asthma due to a reduced growth in childhood and early adulthood, and/or accelerated loss of lung function in adulthood (80). An American 25-year follow-up study of 2,552 participants found that the loss of lung function was already present before the age of 25 in patients having an asthma diagnosis before age 25, while the loss of lung function in people with asthma onset after age 25 was primarily attributable to an accelerated loss in adulthood (81).

A Danish study found that the loss of lung function in patients with asthma was 16 mL/year higher than for people without asthma (82), who are usually assumed to have a loss of 18–26 mL/year (83, 84). However, data suggest large individual differences in annual loss of lung function in patients with asthma. There is currently a shortage of biomarkers that reliably identify patients (children or adults) at risk of excessive lung function decline, yet increased bronchial inflammation or symptom burden are identified as risk factors. These include bronchial hyper-reactivity, frequent exacerbations, persistent symptoms, asthma onset in adulthood, and continuing high FeNO (despite treatment) (85). Due to the abovementioned large individual annual variation in FEV1 measurements over time, a clinically significant decrease in FEV1 cannot be reliably assessed in time periods shorter than 3–5 years (86, 87).

Patients with asthma with a low FEV1% of predicted value are at an increased risk of developing irreversible airway obstruction with time (88) which in turn is a risk factor for increased morbidity (89), increased mortality (90), accelerated loss of lung function (87), and increased number of exacerbations (87).

### How frequently should FEV1 be measured?

FEV1% of predicted value is considered a prognostic marker for exacerbations frequency and the risk of development of irreversible airway obstruction (91). GINA guidelines recommend that lung function is measured at diagnosis and onset of controller therapy and after 3 months of controller therapy (10). This aids in clarifying the risk of exacerbations and irreversible airway obstruction and the optimal FEV1. Recommendations for subsequent FEV1 measurements are less clear due to the between-visit variability of FEV1, limiting its use in adjusting asthma treatment in clinical practice (10).

## Indicator 5: verification of the asthma diagnosis

### Introduction

There is considerable uncertainty associated with the asthma diagnosis because there is no ‘gold standard’ for the disease. The risks of under-diagnosis and over-diagnosis are clinically relevant (5, 92). An incorrect diagnosis implies a risk of over-medication and illness behaviour while under-diagnosis results in increased morbidity. A correct diagnosis not only reduces the risks of over-treatment and under-treatment but also seems to affect patients’ understanding of their disease: patients with ‘objectively’ demonstrated asthma were more likely to adhere to their controller medications compared with patients without objectively demonstrated asthma (93).

### Asthma diagnosis in adults and children over 5 years

In accordance with the GINA guidelines, the asthma diagnosis consists of two steps: The first is identification of symptom characteristic of asthma and the second is proof of variable airflow obstruction. The latter requires serial measurement of FEV1 or, if not available, PEF before and after reversibility test (SABA, ICS, or oral corticosteroid), provocation test (exercise, methacholine, mannitol, hypertonic saline, eucapnic hyperventilation), or completion of PEF diary over several weeks. GINA recommends that the asthma diagnosis is objectively confirmed but accepts that an asthma diagnosis is provided in the presence of characteristic symptoms alone.

The individual tests possess different diagnostic properties. Due to the lack of a gold standard for measurement of asthma, it is challenging to compare tests directly, but evidence for each of the tests is emerging. The diagnostic properties of widely used asthma tests in patients referred from primary care physicians for evaluation of suspected asthma at a respiratory specialist unit were compared in a recent study (94, 95). The four tests included beta-2 reversibility >12%, PEF variation >20%, mannitol provocation, and methacholine provocation. This study underlines that no single test has the optimal diagnostic strength, and that highest sensitivity and specificity is obtained with provocation test with methacholine and mannitol, respectively.

## Indicator 6: allergy testing

### Introduction

According to GINA, allergen exposure in sensitised individuals and in patients with allergic rhinitis is considered to be a potential modifiable risk factor for increased severity of asthma in both children and adults. GINA guidelines recommend clarification of the presence of allergy in adults as part of risk stratification for poor asthma outcome, while guidelines in children are less clear. Clarifying a possible allergic component helps to clarify the presence of preventive asthma triggers as well as allergic non-asthmatic comorbidity requiring other therapeutic interventions.

### Sensitisation and asthma

Sensitisation to both food and inhalant allergens is very common among children and young adults. A previous study found the prevalence of sensitisation to at least one of the most common allergens to be 43% in children aged 0–3 years, and as high as 78–80% in children aged 4–16 years (96). For several decades, numerous longitudinal studies have attempted to describe the natural course of asthma during childhood to adulthood, including attempts to clarify the importance of sensitisation in children with respiratory symptoms. A recent review of the most frequently cited studies concluded that sensitisation against a number of inhalant allergens is associated with persistent asthma symptoms in children (97). At the same time, only a small number of sensitised individuals actually develop symptoms defined as asthma (97, 98). Longitudinal studies report associations between early sensitisation against inhalant allergens and persistent respiratory symptoms (99–103). Simpson et al. has shown that multi-sensitisation in early age was a strong risk factor for later development of asthma (104).

In adults, Osborne et al. conducted a prospective 30-months follow-up study and reported that a positive skin prick test for cat or dog dander was associated with an increased risk of asthma exacerbation with a relative risk of 1.49 (95% CI 1.1–2.0; 76). Another study reported that sensitisation to house dust mites was associated with emergency room visits with exacerbations, and increasing titer of house-dust-mite-specific IgE was associated with a further increase in the risk of hospitalisations (105).

It seems that in children with airway symptoms, sensitisation to allergens is a strong risk factor for persistent asthma, although not all children with sensitisation and airway symptoms develop asthma. Aeroallergen sensitisation and associated humoral and cellular immunological apparatus possibly interact with pro-inflammatory co-factors in the environment, including irritants and viral-induced infections in childhood, resulting in pro-asthmatic inflammation (98, 106).

### Allergy, rhinitis, and asthma

Allergologic workup can be used to determine whether specific allergens affect the patient’s symptoms, and influence asthma disease progression, variation, and treatment. In turn, this determines how the patient should be optimally advised and treated (elimination, allergen-specific immunotherapy, anti-allergic drugs).

An association between food allergy and asthma has been shown, and in combination these increase the risk of severe reactions and death (107). Rhinitis (allergic or non-allergic) is present in 60–80% of asthma patients, and accompanying rhinitis is a positive association with asthma symptom severity and the risk of exacerbations (108).

In patients with severe asthma, the elucidation of an allergic component is of differential diagnostic relevance (109). In patients suspected of occupational allergy, clarification is of major importance to identify symptom-triggering allergens, affecting the patients’ future employment (110).

### Prevention

Allergen elimination is a cornerstone in medical allergology, as primary, secondary, and tertiary prophylaxis. Allergen-reducing interventions as secondary prevention (to avoid symptoms in sensitised subjects) have been examined in several large-scale studies all showing that it is possible to reduce indoor allergen concentration, yet the results regarding the clinical impact have been contradictory (111, 112). In the Manchester Asthma Allergy Study, it was found that the intervention group, in which the concentration of dust mites was significantly reduced, had an increased risk of allergy against house dust mites (111). There is no sufficient data available to recommend interventions to regulate the exposure to common allergens with the purpose of preventing allergy and asthma, both with respect to prenatal and postnatal exposures.

A reduction in allergen exposure as tertiary prevention (treatment of clinical symptoms) has also been discussed. Some allergens can effectively be avoided by a sensitised individual including animal dander, while outdoor allergens such as pollens are almost impossible to eliminate without extensive intervention. The effect of different interventions in patients with house dust mites was examined in a systematic review that included 54 studies and found no effect on peak flow, symptoms, or medication use (113). A meta-analysis that examined the effect of impermeable sheets showed no significant effect on the relative risk for sensitisation against house dust mites, wheezing, asthma, allergic rhinitis, or allergic dermatitis (105). Other clinical studies indicate that tertiary prevention may have a role in the treatment for asthma with verified allergy: improvement in bronchial hyperactivity and symptoms control after interventions consisting of a clean environment without dust mites (111, 114–116). Yet, it remains unclear which interventions are most effective. According to Custovic et al., the impact of allergen-reducing interventions on several levels in meticulously selected patients has not been clarified (117).

In conclusion, it seems reasonable to attempt allergy avoidance as tertiary prevention in selected patients on the basis of individual symptoms, sensitisation patterns, and exposures.

### Allergen-specific immunotherapy

Patients with allergy and asthma may benefit from allergen-specific immunotherapy (SIT). In contrast to elimination and conventional medical therapy, SIT modifies disease course so that risk of asthma is reduced in non-asthmatic children and adults treated with SIT for allergic rhinitis (118). In patients with allergic asthma, SIT reduces asthma symptoms and the need for controller therapy (118–120). SIT is not recommended in patients with uncontrolled asthma, but new data suggest that SIT in combination with omalizumab treatment may improve the clinical effect and reduce the risk of severe side effects (121).

The international guidelines on rhinitis suggest that SIT is considered in severe seasonal rhinitis, and in moderate to severe perennial rhinitis (122). In Scandinavia, the relevant aeroallergens are birch pollen, grass pollen, and house dust mite allergen (dermatophagoides pteronyssinus and farina; (123), but the pattern of allergen sensitisation differs between geographic areas.

Treatment with SIT is currently limited by therapy factors (few different vaccines available, costs, and long duration of treatment), disease factors (multi-sensitisation, co-existence of allergic and non-allergic asthma, and non-responders), and patient factors (adherence, costs, inability to reduce other asthma triggers including allergen avoidance; (124).

It remains unknown if all relevant patients with asthma are also offered SIT, but with respect to patients with allergic rhinitis, only a minority of highly symptomatic patients is offered SIT (125). New measures such as sublingual immunotherapy, governmental refunding, increased awareness in primary care, and careful selection of patients might improve the use and success of SIT (126).

## Indicator 7: smoking

### Introduction

More than 65 years ago, Doll and Hill first reported that active smoking is associated with significant health risks including lung-cancer development (127), and in subsequent decades, the harmful impact of passive smoking has also been highlighted. The underlying pathophysiological mechanisms include a combination of changes in the pulmonary immune system, pro-inflammatory response, and increased oxidative stress (128, 129).

Over the past decades, the proportion of smokers has declined, both among adolescents and adults (130) and in children exposed to passive smoking (131). Since 2003, 177 countries have joined the WHO Framework Convention for Tobacco Control with the objective of gaining control of the global consumption of tobacco and thereby reducing mortality and comorbidity associated with smoking. The effect of this law enforcement in adults was studied in a meta-analysis in 2012 which concluded that the legislation enforcement has been associated with a significant lower risk of asthma and respiratory infections (132). In children, anti-smoking laws have been associated with a significant reduction in the number of asthma-related hospital admissions (133).

### Passive smoking in children

The prevalence of passive smoking in children and adolescents has generally been declining over the past decades, but a recent report from the United States shows that the fall is exclusively observed in children without asthma, while children with asthma continue to be exposed to passive smoking to the same extent (134). In addition, parents of children with asthma tend to under-report the amount of second-hand smoke their children are exposed to (135).

Two systematic reviews from 2014 concluded that exposure to prenatal and postnatal passive smoking increases the risk of asthma diagnosis, asthma symptoms, use of beta-2 agonists and leukotriene-receptors, and exacerbations in children (136, 137). The included studies were, however, of varying quality, often had different aims, and used different definitions of asthma (136, 137). Furthermore, it is challenging to ascribe the isolated impact of prenatal and postnatal exposure, respectively, since parents who smoke during pregnancy, often smoke after birth (138). The conclusions from the reviews were supported by a meta-analysis that reported an association between passive smoking and doctor-diagnosed asthma in children with a pooled OR 1.32 (95% CI 1.23–1.42; (139). Recently, published studies describe similar associations of an increased risk of asthma outcomes or reduced lung function after exposure to passive smoking in children (140–148).

The association between exposure to passive smoking in childhood and sensitisation to aeroallergens is less clear. Ciaccio et al. failed to find an association (149). A systematic review from 2014 found an increased risk of sensitisation in pre-school children based on a meta-analysis of 6629 individuals, although the effect was modest (OR=1.20, 95% CI: 1.05–1.38 for specific IgE and OR=1.30, 95% CI: 1.05–1.61 for skin prick test). The effect in children older than 7 years was not significant (150).

### Smoking in adolescence and adulthood

The impact of smoking in adolescents is poorly studied. Lawson et al. described a link between passive smoking and the onset of asthma in adolescence among 956 Canadian subjects aged 12–18 years with no history of asthma diagnosis but found no significant association between active smoking and the development of asthma in adolescence (151). A cross-sectional study in 2009 that surveyed 1,492 Korean adolescents aged 15–16 years found that active smoking was associated with wheezing within the past year (OR=4.5 95% CI 1.5–13.2), and wheezing with physical activity in the past year (OR=8.7 95% CI 3.7–20.9; 152).

In adult patients with asthma, active smoking is associated with more pronounced symptoms, reduced asthma control, and reduced efficacy of treatment with ICS (153), and they experience frequent exacerbations, episodes of wheezing and nocturnal symptoms, and higher mortality (154). There is evidence for more frequent asthma-related admissions in adults who are current and former smokers compared with never smokers (155).

There is substantial evidence of the harmful impact of active smoking in asthma patients, but whether smoking is associated with an increased risk for the onset of asthma in adults is controversial. Many cross-sectional studies have shown a link between smoking and the onset of asthma, but these studies cannot explain a causal relationship. Several cohort studies support this, while others have found no relationship, and one describes a lower incidence of asthma in current smokers (129, 156). The most recent published study of the association between asthma and tobacco is a large cohort study from the United States, which followed 46,182 people without asthma diagnosis at baseline and with an average follow-up of 14.7 years. This study found a clear link between active smoking and the development of asthma, as well as an increased risk of developing asthma in non-smokers exposed to second-hand smoke (129).

### Long-term impact of smoking on asthma

The long-term consequences of active smoking in asthma patients have not yet been fully clarified, and present conclusions are contradictory. Some studies describe a more rapid decline in lung function in adult asthma patients who smoke, and early development of COPD in some (157), while studies from the European Community Respiratory Health Survey do not support this (158). However, there are studies that clearly show that smoking cessation is associated with improvement in lung function, slower decline in lung function, and better asthma control (154, 158). It has further been shown that smoking accelerates the decline in FEV1 in patients with asthma, and that treatment with ICS can reduce this decline in FEV1 in asthmatics who smoke (159, 160).

## Indicator 8: height and weight

### Introduction

For several years, it has been debated whether systemic treatment with inhaled steroids affects the growth (height) in children. In 1980, suspicions were based on individual cases, while early trials and meta-analyses failed to detect a significant effect on height development (161, 162). However, in subsequent years, several short-term studies have reported a significant reduction in height development (growth) after a few months of treatment primarily in pre-pubertal children (163). Consequently, the US Food and Drug Administration decided that the patient information leaflets of inhaled steroids should include a clear warning that the treatment could potentially constrain growth (height; 164).

### Evidence of an effect on height after treatment with ICS

In a recent Cochrane review of 25 randomised trials with a total of 8,471 children with mild to moderate asthma, who were treated with ICS over a period of 3 months to 4–6 years, it was found that the regular use of ICS in low to moderate dose over a 1-year period reduced the growth rate by 0.48 cm/year (95% CI −0.65 to −0.30) and the absolute growth by 0.61 cm (95% CI −0.83 to −0.38) compared with a control group treated with placebo or non-steroid. The reduced growth was most pronounced in the first year, after which the effect was less pronounced (165).

In 2014, the dose dependence of treatment with ICS and the reduced length growth was examined in another Cochrane review, which reviewed 10 randomised trials that included a total of 3,394 pre-pubertal children with mild to moderate asthma who were treated with ICS monotherapy or ICS in combination with LABA. Treatment with a low to moderate dose (200 µg HFA beclomethasone equivalent) was associated with a reduction in the growth rate by 0.20 cm/year (95% CI 0.02–0.39) after 12–52 weeks of treatment compared with those treated with a low dose (50–100 µg HFA beclomethasone equivalent; (166).

Whether ICS treatment is given fixedly or intermittently is also important as highlighted in yet another Cochrane review from 2013. This meta-analysis, included 278 pre-school children and 330 school-age children, and showed a significant reduction in longitudinal growth by 0.41cm (95% CI 0.13–0.69) in children treated with regular daily ICS (budesonide or beclomethasone) of 44–52 weeks, compared with intermittent therapy over 44–52 weeks (167).

It is unclear whether the final height in adulthood is influenced by long-term treatment with ICS as present studies with follow-up to adulthood describe conflicting results. The largest study, The Childhood Management Programme, examined the final height of 943 of the 1,041 participants aged 5–13 years with randomised treatment with budesonide 400 µg/day, 16 mg nedocromil/day or placebo with mean treatment duration of 4.3 years. The study found an average height reduction of 1.20 cm (95% CI −1.90 to −0.50) in the final height compared with placebo (168). In contrast, Agertoft et al. found that normal final height was achieved in 142 Danish children with asthma who were treated with budesonide in a mean dosage of 412 µg/day over an average period of 9.3 years (169).

### Obesity and asthma in children

It has been shown that children with asthma are at a higher risk of developing obesity compared with children without asthma (170). It is unclear whether child obesity precedes the asthma disease, or overweight occurs as a result of inactivity due to respiratory symptoms, or if overweight is an asthma phenotype as several longitudinal studies have reported that children who were overweight at inclusion or became obese during the study had an increased risk of asthma symptoms and asthma diagnosis (171). In a meta-analysis from 2013, including six studies with a total of 18,760 children aged 6–18 years, it was shown that child obesity increases the risk for the onset of asthma (172).

Obesity in children with asthma is associated with negative health consequences, including increased risk of hospitalisation, longer convalescence during hospitalisation for exacerbation, and more pronounced symptoms compared with normal-weight children with asthma (173, 174). Pharmacological treatment has also been shown to be compromised with a poorer response to ICS and low-dose theophylline in children with obesity, although there was a slightly increased response to montelukast (171). In children with asthma, weight loss interventions may improve lung function, asthma control, and quality of life (175–178).

### Obesity and asthma in adults

The link between obesity and the prevalence of asthma was clearly shown in a meta-analysis in 2007 including 333,102 adults, and the result showed a dose–response relationship between increasing body mass index (BMI) and the incidence of asthma (179). In adults with asthma, obesity is associated with a range of adverse asthma outcomes, including more pronounced symptoms, increased absenteeism from work, increased consumption of reliever medication, a lower probability of good asthma control, poorer response to anti-asthmatic treatment, and increased risk of asthma exacerbations that require treatment with systemic steroids (180, 181).

Some data suggest that asthma in obese subjects should be perceived as a distinct phenotype where symptoms remit after weight loss, including improvements in lung function, better symptom control, lower need for medication, and fewer admissions (180).

The pathophysiological basis for the association between obesity and asthma is not yet established, but existing theories are based on the fact that obesity is a pro-inflammatory state. It has been shown that isolated obesity without accompanying comorbidity is associated with a slight systemic inflammation and increased oxidative stress. These relationships are thoroughly examined in two recent reviews of Ali et al. (180) and Sutherland et al. (182). Therapeutic weight loss in adults with asthma was reviewed in a Cochrane review in 2013 and concluded that weight loss can reduce asthma symptoms and use of reliever medication, but the studies also had significant methodological limitations at high risk of bias and deficient data to draw firm conclusions (183).

## Indicator 9: acute hospitalisation

### Introduction

Asthma is a leading cause of acute hospitalisation among children. In an inter-European survey of 2000 randomly sampled patients (of which 753 children and 2050 adults had asthma), researchers found that 18% of children and 11% of adults with asthma had at least one contact to the emergency room within the preceding 12 months (184). In addition, 7% of the entire included asthma population had been hospitalised (defined as a minimum of one night) due to asthma within the preceding year. In a Portuguese population survey from 2015 of asthmatic children and adolescents aged between 6 and 17 years, 27.7% had at least one hospitalisation of more than 24 h during their life (185). Of those who had contact with the emergency room with asthma symptoms, it was estimated that almost 10% will subsequently be hospitalised (186, 187).

The average hospitalisation duration in 2003 due to asthma was estimated to last 0.26 days in adults (188) and 2.6 days in children (189). However, figures vary considerably from study to study and a Danish study including 100 adults with exacerbations concluded an average time in the emergency room of 10.7 h, while an in-patient visit took on average 3.6 days (190). The subsequent cost of hospitalisations due to asthma is high (188).

Emergency room visits are most common in asthma patients with uncontrolled disease, but also occur among people with otherwise mild or well-controlled asthma (191–193). There are large individual differences in the number of calls to the emergency room with asthma, some asthma patients are rarely or never seen in anything but the primary sector, others have more annual unplanned contacts (194, 195). Women are over-represented in the group of persons requiring hospitalisation for asthma (196).

### Exacerbations

In most drug studies in which admissions or contacts to the emergency room due to asthma are detected, the hospitalisation/emergency room visit is rarely the primary endpoint, but is included as part of the definition of an ‘exacerbation’. The criteria for exacerbation and severity of these vary somewhat from study to study (197), but in a joint working document of the American Thoracic Society and European Respiratory Society published in 2009 (197), an exacerbation is defined as: ‘episodes that are troublesome to patients, and that prompt a need for a change in treatment’. The document further describes in detail how moderate and severe exacerbations are defined in children and adults.

### Causes of exacerbations

Exacerbations are mostly triggered by well-known risk factors such as viral infection [up to 85% of exacerbations in children and 60% in adults, particularly rhinovirus (198), allergens or pollution (199)]. The main risk markers for exacerbation in both children and adults include: previous severe exacerbation (OR=6.33 in adults) (200), overuse of SABA and inadequate treatment with ICS, low FEV1 (79), socio-economic factors, pregnancy (201), tobacco exposure (202), irreversible airway obstruction, eosinophilia (203), obesity, severe rhinosinusitis (204), and hypersensitivity to non-steroidal anti-inflammatory drugs (205, 206). Most of these risk factors are listed in the GINA guidelines and therefore frequent evaluation of these risk factors is recommended.

### The impact of exacerbations

The ultimate consequence of exacerbation in rare cases is death (207). In children, the most common cause is the absence of preventive treatment with ICS, besides poor adherence, severe atopy, and food allergy (207, 208). Exacerbations reduce the quality of life (209) and lead to an increased loss of lung function. In a cohort study, it was found that asthmatics with at least one severe exacerbation per year had an increased loss in FEV1 of 30.2 mL/year (210).

In an American study of children and adolescents with and without asthma, asthmatics had more annual sick days than non-asthmatics (9.2 vs. 7.9 days) (211). The same difference was also found in another US study in which asthmatic children had an average of two more days of absence from school compared with non-asthmatics (212). Figures from Sweden published in 2004 showed that 34% of children and 13% of adults had at least one day of absence due to asthma (213). In line with this, a study published in 2002, including almost 2,600 people with asthma, found an average absence per year due to asthma of 2.1 days (214). Finally, in Denmark, a study estimated the production loss due to asthma sick leave to be more than US$40 million annually (188).

## Summary and discussion of the main findings

DNDA is a clinical quality database with the overall aim to continuously collect data on all patients with asthma, who are diagnosed, monitored, or treated for the disease anywhere in Denmark. DNDA links information from three nationwide registries of administrative records in the Danish health care system providing a tool to continuously collect the data on all patients with asthma (6). The steering committee of the DNDA has chosen a range of indicators that constitute the quality parameters. In this article, the literature supporting each of these indicators has been presented. In the following, a brief summary of the evidence is presented along with a description of the nine quality indicators in the DNDA and the standards for documented quality of asthma management that have been set by the steering committee to form good quality. The standards expressed as a percentage can be high or low depending on whether the direction of the improvement is upwards or downwards, respectively. The standards may be modified continuously if new evidence emerges or if the steering committee sets new goals for good quality. The standards described here therefore constitute an initial attempt that will be used in the first report of the DNDA to be published in 2017. Ongoing revisions to the indicators or/and the standards can therefore be expected in future reports.

The first quality indicator in the DNDA is ‘control visits’. There is general consensus that asthma control visits are important elements in asthma care to ensure symptom control and assessment of risk factors for exacerbations. It is known that a rigorous approach aiming for asthma control gives a near-normal quality of life and reduces the risk of exacerbations (7). However, there remains no clear recommendation regarding the frequency of asthma control visits. To ensure a reduction in symptoms, normalisation of FEV1, and to clarify the best score on spirometry, it is reasonable to complete control visits 3 months and 12 months after diagnosis and initiation, respectively, or titration of anti-asthmatic therapy. The optimal frequency of subsequent asthma control visits differs among asthma patients based on individual needs. We found no evidence that asthma health care visits exclusively for monitoring lung function affect symptom control. Adherence and inhalation techniques are essential for achieving asthma control and should be assessed and trained regularly (20, 21). It may be appropriate to follow patients with frequent exacerbations or risk of exacerbations closely in order to intervene on modifiable risk factors.

Despite the conflicting findings in the literature, the steering committee has decided that indicator 1 in the DNDA will quantify the proportion of asthma patients, who are monitored and registered annually with a control visit. Initially, the standard to be met has been set to ≥80% but can be changed in case new evidence regarding control visits emerges.

The second and third indicators in the DNDA are related to pharmacological treatment. Available studies show that asthma is underdiagnosed and undertreated in both children and adults (5, 31, 32, 34, 35). Undertreated asthma leads to reduced asthma control and increases the risk of exacerbations, hospitalisation, and increased health care costs, emphasising the need for identifying these individuals (40, 41). Because reasons for the inadequate treatment of asthma include the health professional’s lack of adherence to clinical guidelines, it is reasonable to assess the pattern of prescriptions of anti-asthmatic therapy.

Since establishment of the Danish Civil Registration System in 1968, all citizens of Denmark have a unique Personal Identification Number (CPR number). All medical, social, and other administrative public records use this unique number to identify citizens: thus, this number allows accurate linkage between registers. The Danish Registry of Medicinal Product Statistics has collected data on prescription drugs since 1995 and contains information on patients’ CPR number prescription date, specific anatomical therapeutic codes of the type of drug, and amount in dosage numbers. However, this registry has not yet been used for systematic monitoring of treatment of asthma. Prescription data on SABA can be used as a surrogate marker of poor asthma control and predict asthma exacerbations, but we found large differences of the reported SABA prescription associated with the negative outcomes (48–53, 55). Based on the available literature, it has been decided by the steering committee to let indicator 2 in the DNDA evaluate the proportion of asthma patients, who are currently not treated with ICS despite a high consumption of (>600 DDD per year) SABA. The standard has been set to ≤10%. Present GINA guidelines do not support the use of LABA or LABA as monotherapy or LABA/LAMA in combination, making these prescription patterns possible surrogate markers of quality of asthma treatment also. It has been decided that indicator 3 in the DNDA will evaluate the proportion of asthma patients who are using LABA, LAMA, or LABA/LAMA without a concurrent consumption of ICS. The standard has been set to ≤1%.

Lung function constitutes indicator 4. Spirometry is a standardised and validated tool to measure lung function and is widely used in the assessment of asthmatics. However, FEV1 only weakly reflects the amount of the symptoms or underlying airway inflammation. FEV1 is a prognostic marker for the number of exacerbations (particularly FEV1 <60% predicted), development of irreversible airway obstruction and death (77–79). To be able to monitor changes in FEV1 over time, repeated measurements with the assessment of the patient’s best FEV1 early stage is required. Once the individual optimal FEV1 is assessed, it is recommended to reassess regularly, but because of individual variations in FEV1 we have found no evidence for progressive monitoring with FEV1 measurements more than every 3–5 years in adults. Despite this fact, the steering committee has decided that indicators 4a and 4b in the DNDA will measure the proportion of patients who are currently treated for asthma in out-patient clinics who have their lung function measured annually. The standard has initially been set to 98%. For incident patients, the lung function should be measured within 6 months before the first asthma diagnosis or 6 months after the first diagnosis, and for patients aged 6–17 years, the standard has been set to ≥98%, whereas for patients aged 18–45 years, the standard has been set to ≥80%.

Verification of the asthma diagnosis constitute indicator 5. We found evidence supporting the fact that an objective confirmation is important to ensure correct diagnosis, treatment, and follow-up. Furthermore, a recent, yet unpublished, study indicates that asthmatics that did not receive a thorough and objectively demonstrated asthma diagnosis are less likely to use the prescribed anti-asthmatic treatment (93). There are several different tests that can be used in the diagnosis of asthma, and the various diagnostic tests have overlapping diagnostic patterns reflected by differences in the sensitivity, specificity, positive predictive value, and the negative predictive value. The steering committee has decided that indicator 5 in the DNDA will quantify the proportion of incident asthma patients who have a reversibility test, a provocation test, or an exertion test performed within 6 months before and 6 months after the initial diagnosis for asthma. The standard has been set to ≥80% for all patients.

Indicator 6 concerns allergy testing. Allergic sensitisation is common in patients with asthma, and sensitisation against inhalant allergens in children with asthma is associated with persistent asthma symptoms (215, 216). Allergy testing in individuals with asthma is relevant for risk stratification of patients, as sensitisation against inhalant allergens is associated with an increased risk of exacerbations and because allergic comorbidity is shown to be associated with more pronounced symptoms of asthma. It is always appropriate to determine whether the indications for specific immunotherapy are fulfilled. Allergen elimination has been debated for centuries because of conflicting results, but may be appropriate in selected cases. Indictor 6 in the DNDA will evaluate the proportion of incident asthma patients who have a skin prick test or quantification of specific IgEs performed within 6 months before and 6 months after the initial diagnosis. The steering committee has decided that for patients aged 6–17 years, the standard to be met is ≥98%, whereas for patients aged 18–45 years, the standard has been set to ≥80%.

Indicator 7 is related to smoking. There is evidence that passive smoking in childhood is associated with the development of asthma, and in children with asthma associated with more pronounced symptoms, greater use of asthma medications, and higher risk of exacerbations (217–221). Both active and passive smoking in adults increases the risk of developing asthma, and active smoking in adults with asthma is associated with more pronounced symptoms, poor asthma control, an accelerated decline in lung function, and reduced efficacy of treatment with inhaled steroids, as well as more frequent exacerbations and higher mortality (152–159, 222).

Intervention in clinical practice has been shown to be effective and should be practised. Both passive and active smoking should be discussed with the patient by relevant clinical contacts both in patients with and without asthma. Smoking legislation reduces tobacco-related disease and smoking-related hospitalisations (132, 133). The steering committee has decided to let indicator 7 in the DNDA quantify the proportion of asthma patients who are inquired about their smoking status at least once a year. The standard for all patients has been set to ≥80%.

Indicator 8 concerns weight and height measurements. This indicator is included because of evidence that treatment with ICS may result in a modest dose-dependent reduction in longitudinal growth in children 1–2 years after the initial treatment is started, but it is unclear whether the final height is affected, as current data are conflicting (163, 164).

Obesity in children and adults with asthma is associated with significant adverse effects on asthma disease and weight loss should be recommended in the treatment of asthma in both obese children and adults. BMI should therefore be determined at a minimum when the diagnosis is made (171–174). It has been decided to include two indicators on height and weight measurements in the DNDA. Indicators 8a and 8b will evaluate the proportion of asthma patients who have their height and weight measured, respectively, at least once a year. Indicator 8a will only be evaluated in patients aged 6–17 years. The standard has been set to ≥80%.

Finally, indicator 9 in the DNDA is related to hospitals admissions. We found evidence that admissions to the emergency room and/or hospitalisations due to asthma are frequent.

Exacerbations are linked to increased risk of death, quality of life, increased loss of lung function, and increased costs for health. In the GINA guidelines, it is recommended that the risk factors for exacerbations are regularly evaluated in all patients with the purpose of preventing hospital admissions. In the DNDA, indicators 9a and 9b will quantify the proportion of patients with asthma currently treated who end up in the hospital with an acute admission for a duration of ≤1 day and >1 day, respectively. No specific standard has been set.

The steering committee of the DNDA believes that a continuous, systematic collection of relevant clinical information is needed to improve asthma treatment in Denmark. This includes targeting the use of diagnostic tools in suspected asthma cases and standardising treatment of the same level of the disease, reducing over-treatment and under-treatment, and ensuring better continuity in the follow-up of asthma patients. The DNDA will potentially hold a unique set of information, including development in lung function, use of medication and clinically relevant endpoints like exacerbations in an asthma population that includes all sectors of the nationwide health care system in Denmark.
